# Immunohistochemical detection of hTERT in urothelial lesions: a potential adjunct to urine cytology

**DOI:** 10.1186/1742-6413-3-18

**Published:** 2006-08-10

**Authors:** Walid Khalbuss, Steve Goodison

**Affiliations:** 1 Dept. of Pathology, University of Florida, Jacksonville, FL, USA; 2 Dept. of Surgery, University of Florida, Jacksonville, FL, USA

## Abstract

**Background:**

Urine cytology has a critical role in evaluation for bladder carcinoma. Due to the low sensitivity of this technique, ancillary modalities such as the detection of markers of malignancy by immunochemistry are desirable. Promising factors in this context are components of the human telomerase enzyme complex. Telomerase repairs and extend telomeres, which when eroded beyond a critical limit trigger a senescence checkpoint. Accordingly, while absent in normal somatic cells, telomerase activity has been detected in the great majority of malignant tumor specimens tested, and so has potential value for the recognition of malignant cells in clinical specimens.

**Methods:**

In this study, we investigated whether the immunohistochemical detection of the catalytic subunit of telomerase (hTERT) can aid cytology in the diagnosis of bladder lesions. Findings from the retrospective evaluation of over 100 cell blocks, including urine sediments from confirmed malignant and benign conditions, were compared with routine urine cytology data.

**Results:**

The presence of hTERT protein was indicative of the transformation of urothelia to a malignant phenotype. Nucleolar hTERT was expressed in 27 (93%) of 29 samples obtained from patients with confirmed primary bladder cancer. Conversely, hTERT was detectable in only 3 (0.8%) of 39 samples from benign conditions. The hTERT assay showed higher diagnostic sensitivity (84.8%) than published urine cytology data (~65%) for confirmed bladder carcinoma, however, the hTERT assay was less specific than cytology (65.2% vs. ~95% respectively).

**Conclusion:**

As a highly sensitive marker, immunohistochemical hTERT detection in urine sediments represents a reliable adjunct to cytology in the accurate diagnosis of urothelial neoplasms.

## Background

The diagnosis of suspicious bladder lesions is, in part, dependent on the demonstration of atypical cells in the cytological examination of voided urine or bladder washings. However, the relatively low diagnostic sensitivity of urinary cytology warrants the development of improved non-invasive diagnostic techniques [1]. Furthermore, one of the major problems in daily cytology practice is to distinguish benign/reactive cells from malignant cells. Additional techniques, such as immunocytochemistry and flow cytometry, may provide significant help in this differential diagnosis. A number of antibodies directed against specific cell type antigens have been used in urine cytology to enhance the cytological diagnosis, including NMP22, cytokeratin 20 and human complement factors, but the results with cytological preparations have been conflicting [2-4]. No single diagnostic technique alone is sufficient to establish the diagnosis in all cases, and the search for an accurate tumor marker that reliably confirms urothelial malignancy remains a challenge. Promising factors in this context are components of the human telomerase enzyme complex. The human telomerase reverse transcriptase (hTERT) protein is the catalytic subunit of the telomerase holoenzyme which maintains chromosomal telomeres [5]. Telomeres are the non-coding termini of eukaryotic chromosomes and function to stabilize and maintain chromosomal structure. However, telomeric DNA is lost at each cell division as a result of the inability of DNA polymerases to replicate the 5' end of linear DNA [6], and erosion of these sequences beyond a critical point is thought to signal cell cycle arrest and entry into cellular senescence [7]. The major mechanism of telomere repair or maintenance is mediated by the enzyme telomerase [5]. A close association between the activation of the telomerase enzyme and cellular immortality has been established, and the presence of functional telomerase enables cells to be capable of extended proliferation or to become immortal, and in concordance with this hypothesis, telomerase activity has been detected in the great majority of malignant tumor specimens tested [8,9]. The enzyme is undetectable in normal somatic cells; therefore, the detection of telomerase activity in human tissue samples has value for the recognition of malignant cells in clinical specimens [10].

For detecting telomerase activity in a tissue specimen the TRAP assay is a relatively sensitive and specific method, but it can be used only on fresh tissue extracts and offers no information at the cellular level [11]. Expression of hTERT mRNA is very closely associated with telomerase activity in human tumors and can be detected by RT-PCR [12]. However, this approach also does not offer any information at the level of the individual cell and so correlative comparison of molecular data with cellular morphology is not attainable. Therefore, immunohistochemical (IHC) methods of hTERT protein evaluation, which can both detect *and *localize cellular telomerase expression in human tissue would be optimal for the differential diagnosis of cellular material such as serous effusions. Investigators have recently tested commercially available anti-hTERT antibodies in formalin-fixed and paraffin-embedded human tissues by IHC [13,31]. One monoclonal antibody (NCL-hTERT; Novacastra) was sufficiently specific for further investigation in clinical specimens.

In this study, we applied the hTERT antibody to urine sediment cytology samples prepared as paraffin sections of cell blocks. We evaluated over one hundred cell blocks for hTERT immunoreactivity, and compared the findings with available conventional cytology and biopsy pathology information. When present, the expression of hTERT protein was localized to the nucleoli of urothelial cells, and hTERT expression positively correlated with urothelial cell neoplasia. Whilst conventional cytology plays a pivotal role in the diagnosis of bladder cancer, for difficult cases, in which ancillary information is necessary, the use of immunohistochemical detection of the telomerase component hTERT may significantly improve diagnostic accuracy.

## Methods

### Patient specimens

In total, 101 cell blocks that contained various bladder tissue specimens were identified in the archives of the Shands & University of Florida Hospital, Jacksonville, FL. Cell blocks were prepared from urinary sediments collected from patients under investigation for bladder lesions of various types. The urinary sediments were processed into cell blocks using the plasma/thrombin technique [14]. Evaluated specimens were selected according to the cytological diagnosis and included 29 malignant cases, 39 non-malignant cases, and 33 cases of cytological atypia. Tissue biopsy confirmation of malignant and benign conditions was available in 56 cases.

### Urine cytology

Smears of urine specimens from each patient were examined cytologically by standard Papanicolaou staining. All slides were evaluated routinely by an experienced cytopathologist without any prior knowledge of the immunohistochemical findings. Urothelial carcinoma grading and staging were performed according to the World Health Organization criteria.

### Immunohistochemistry

**Figure 1:**
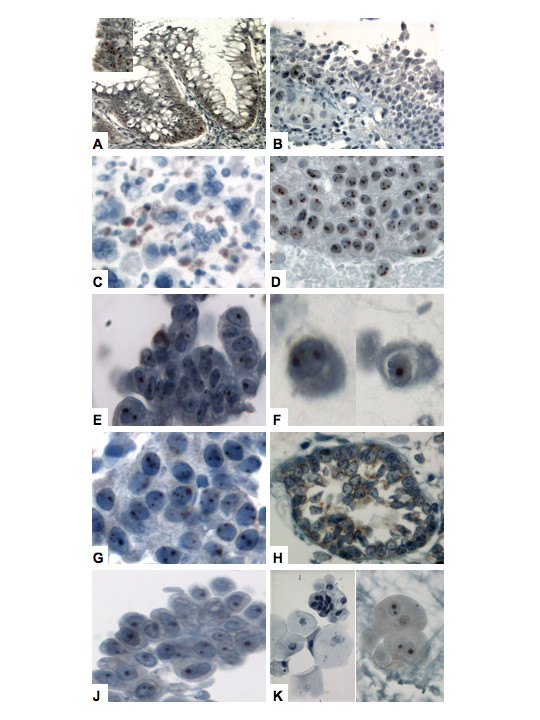
Expression of hTERT protein in human urological tissue. hTERT immunostaining was localized in the nucleolus of urothelial cells. Formalin-fixed, paraffin-embedded bladder tissue biopsy (A, B) and urinary sediments (C-K) were analyzed as described in 'materials and methods' (original magnification ×200): (A), hTERT expression was generally confined to the lower half of normal colon crypts; (B, inset at higher magnification), bladder tumor biopsy, in which it can be seen that neoplastic cells and proximal, morphologically normal urothelia react with the antibody; (C), example of immunoreactive yeast present in urinary sample; (D), transitional cell carcinoma (low grade); (E), transitional cell carcinoma (high grade); (F), squamous cell carcinoma; (G), carcinoma in situ (CIS); (H), cystitis glandularis; (J), urolithiasis; (K), umbrella cells (left panel shows negative example, right panel shows an immunoreactive example).

The specificity of the antibody has been reported in previous reports [13,31]. Antibody-mediated detection of hTERT was performed using the standard streptavidin-biotin peroxidase complex method. All steps were performed on a Ventana Benchmark XT-BTS automatic immunostainer (Tucson, Arizona). Tissue sections from representative blocks were deparaffinized in xylene and alcohols, and were then placed in 3% hydrogen peroxide/methanol for 5 minutes to block nonspecific background staining due to endogenous peroxidase activity. Antigen epitopes were retrieved (Ventana Benchmark CC1 extended program) by heating to 100°C and reducing heat slowly over a 90 min period at pH 8.0. The primary antibody (NCL-hTERT IgG 2a, Batch # 147508, from Novocastra, Newcastle-upon-Tyne, UK) was diluted 1:25 and applied to the slides for 32 min at 37°C. Secondary antibody incubation, washes and chromogen (3,3'-diaminobenzidine) development were performed at room temperature. Slides were counterstained with hematoxylin, dehydrated, and mounted for microscopical examination. Optimization of conditions was performed with sections of normal colon (hTERT-positive at the base [13] of the crypts) (Figure 1A) and tissue bladder biopsies with urothelial carcinoma (Figure 1B). Negative control slides included a blank control and omission of primary antibody.

### Assessment of hTERT immunostaining

The entire hTERT stained slide was examined for immunostaining of hTERT. Positivity for hTERT expression was evaluated by two independent observers (WK, SG). Nucleolar urothelial cell positivity was used for evaluation of utility in diagnosis. Positivity in non-urothelial cells such as inflammatory cells, lymphocytes, microorganisms, or contaminating debris was duly noted and recorded. The availability of corresponding tissue bladder biopsy material, which is the gold standard for diagnosis or exclusion of urothelial carcinoma, enabled the determination of the sensitivity and specificity of hTERT immunostaining for the detection of bladder cancer. The positive and negative predictive values were determined as follows: *positive predictive *value is equal to the number of truly positive cases identified/total number of cases that tested positive × 100%, *negative predictive value *is equal to the number of truly negative cases identified/total number of cases that tested negative × 100%.

## Results

Optimization of the hTERT immunostaining technique, as described in Materials and Methods, revealed that specific hTERT expression was nucleolar in both proliferatively active normal cells, and in tumor cells. In normal colon crypts, staining intensity was concentrated in the base of the crypt (Figure 1A), as previously observed [13], where the basal cells with proliferative capacity reside. In excised bladder tumor tissue, the expression of hTERT was clearly evident in the multiple nucleoli of neoplastic cells (Figure 1B). Notably, some morphologically normal urothelia adjacent to the tumor border also expressed hTERT, but the immunoreactivity diminished and was lost in normal cells more distal to the tumor (Figure 1B). Diffuse nuclear staining accompanied nucleolar positivity in a few examples, but we did not observe any cytoplasmic staining. Lymphocytes were reactive to hTERT antibody, as were yeast when present (Figure 1C). Yeast is a common contaminant in clinical urine sampling and is known to contain the telomerase enzyme, so the presence of yeast can pose a serious confounding problem in urine-based assays. Using the advantage of histological identification facilitated using a hematoxylin counterstain, we classified patient samples as positive when the presence of specific nucleolar staining was evident in urothelial cells. Patient demographics and tumor characteristics of the samples evaluated are presented in Table 1. The 101 cases came from 53 male patients and 48 female patients. The average age of the patients were 62 years (ranges: 30–91 years). The majority of the specimens were voided urine specimens (61.4%). The majority of the urothelial carcinoma cases were grade III (13 cases) and grade II (8 cases), and one case grade I. The 39 non-malignant cases included diagnoses of urolithiasis, cystitis glandularis and cases with no detectable lesion. We also evaluated 33 cases classified as atypical by cytological examination. Tissue bladder biopsies were available in 56 cases for histological and cytological correlation.

**Table 1:** Characteristics of cases evaluated in the hTERT immunoassay

Sex	M/F	53/48	
Voided urine specimens		62	61.4%
Instrumented specimens		39	38.6%
Positive for malignancy		29	28.7%
	Urothelial carcinoma	25	
	Squamous cell carcinoma	3	
	Small cell carcinoma	1	
Negative for malignancy		39	38.6%
Atypical/undetermined		33	32.7%
Biopsy correlation available		56	55.5%

All types of urothelial cancer expressed hTERT, including transitional cell carcinomas of low grade, high grade, carcinoma in situ (CIS), squamous cell carcinoma (Figures 1D–1G), small cell carcinoma and metastatic adenocarcinoma. Of the 29 cases cytologically diagnosed as malignant and confirmed by biopsy, 27 (93%) exhibited nucleolar hTERT expression (Table 2). One case of high-grade urothelial carcinoma and one case of squamous cell carcinoma did not display hTERT expression. Among the cytologically classified non-malignant cases, transitional cell hTERT expression was detectable in only 3 (<1%) of the 39 samples evaluated. One of the 3 false-positive cases was from a patient with confirmed diagnosis of urolithiasis (Figure 1J). The other 2 cases have no corroborative evidence of bladder disease to date. There were 56 cases (malignant, benign and atypical cytologically) on which we had confirmatory information from bladder tissue biopsy evaluation. Histological examination of tissue bladder biopsy is considered the gold standard for the diagnosis of bladder malignancy. The biopsy correlation data are shown in Table 3. The availability of this data enabled the calculation of the diagnostic accuracy of the hTERT immunoassay. Overall, the hTERT immunoassay demonstrated a positive predictive value of 77.8% and a negative predictive value of 75% in this study. No correlation of hTERT expression was observed with the mode of specimen collection i.e. urine collection through voiding or instrumentation. Of 4 cytologically atypical cases subsequently proven to carry a bladder tumor burden through cystoscopy and biopsy, one case expressed hTERT (Table 3). Thus, in this case hTERT detection may have aided diagnosis, however, of 12 cytologically atypical cases with no evidence of a bladder lesion at the time of sampling, 7 cases exhibited hTERT expression. Notably, 4 of these 7 positive atypical cases were from patients who had a history of bladder cancer, indicative of a possible 'field-effect' which alters the phenotype of the entire bladder urothelia without resulting in an actual focal lesion. Finally, it is noteworthy that umbrella cells were occasionally positive for hTERT expression (Figure 1K). These superficial cells were commonly identified in urine sediments and exhibited hTERT in seven cases, all of which had negative reactive urothelial cells. In this study, cases were only considered positive if urothelia other than umbrella cells exhibited nucleolar hTERT staining.

**Table 2:** Comparison of hTERT immunoassay results with cytological diagnosis.

	hTERT		
Cytological diagnosis	Positive	Negative	Total

Positive	27	2	29
Negative	3	36	39
Atypical	19	14	33
Total	49	52	101

**Table 3:** Evaluation of hTERT immunoreactivity in biopsy confirmed malignant and non-malignant specimens

*Malignant cases*		hTERT		
			
		Positive	Negative	TOTAL
			
Cytological diagnosis	Positive	27	2	29
	Atypical	1	3	4
			
	TOTAL	28	5	33
*Non-malignant cases*		hTERT		
			
		Positive	Negative	TOTAL
			
Cytological diagnosis	Negative/Reactive	1	10	11
	Atypical	7	5	12
			
	TOTAL	8	15	23

## Discussion

Carcinoma of the urinary bladder is the most common malignant tumor of the urinary tract and, after prostatic carcinoma, the second most common malignancy of the urogenital system. Furthermore, bladder carcinoma shows a very high recurrence rate (50–70%) and recurrent disease, when it does occur, is associated in 15–25% of patients with progression to a more advanced tumor stage. Thus, careful and frequent follow-up is of prime importance. Due to factors such as size or localization, the great majority of carcinomas of the urinary bladder are either undetectable with standard imaging techniques, or cannot be definitively differentiated from non-malignant, reactive processes. Thus, the invasive method of instrumental cystoscopy remains the standard detection mode in the clinic. A non-invasive method of comparable diagnostic quality would significantly facilitate the initial diagnosis of carcinoma of the urinary bladder and greatly simplify the follow-up of treated patients. The commonly utilized cytological examination of urine sediments is specific and readily available, but it is not sufficiently sensitive for the detection of the subtle morphologic changes that occur in well-differentiated carcinomas of the urinary bladder. Furthermore, inter-observer variability can create additional diagnostic pitfalls, thus, the need for non-invasive techniques for the diagnosis and follow-up of carcinoma of the urinary bladder remains.

Recent evidence has suggested that the presence of the enzyme telomerase in urine is a potentially useful marker for the early detection of urothelial neoplasia [15-19]. Comparative analyses of non-invasive methods for the diagnosis of bladder cancer have shown that telomerase has the highest combination of sensitivity and specificity with respect to urine cytology and other biochemical marker determinations [16,18-25]. The detection of telomerase activity and the expression of the associated genes has been customarily achieved through the telomere repeat amplification protocol (TRAP) and RT-PCR methodologies respectively. Sanchini et al recently demonstrated the ability of telomerase activity levels in urinary sediments to accurately detect the presence of bladder tumors. A sensitivity of 90% and specificity of 88% was achieved for bladder cancer detection by applying relative cut-off points to a quantitative TRAP assay [26]. Telomerase activity determination by the TRAP assay enables quantitative evaluations to be made, but is vulnerable to contamination by telomerase-positive, non-malignant cell types, such as proliferative stem cells and inflammatory elements [27,28]. Furthermore, because the TRAP assay detects enzyme activity and not simply the presence of the protein, valid findings require the presence of living cells. In native urine, suspended cells are exposed for various lengths of time to destructive substances and conditions including proteases, urea, and acidic pH values [10]. The alternative to the TRAP assay is detection of the structural or encoding RNA components of the telomerase enzyme, primarily the human telomerase RNA component (hTR) and hTERT mRNA [10,21,27,28]. Compared to other urine-based markers, quantitative detection of human telomere components by RT-PCR, particularly the RNA component (hTR), shows acceptable diagnostic accuracy with sensitivities of up to 77% (29). Clearly, telomerase is a promising marker for urothelial neoplasia, but these tests remain several steps removed from becoming a routine procedure which replaces cytology. In contrast, an *in situ *analysis would enable the morphological identification of hTERT-positive cells, and if used as an adjunct to cytology such analyses could be rapidly brought into practice. Good monoclonal antibodies that specifically recognize hTERT have been isolated only recently so few studies have focused on the immunohistochemical evaluation of hTERT in human tissues. In the current study, we demonstrated the feasibility of an hTERT immunoexpression assay on urine sediment samples, and investigated its utility as an adjunct to conventional urine cytology in the diagnosis of bladder carcinoma.

Our study has shown that through the application of a monoclonal antibody, hTERT can reliably be detected in paraffin-embedded solid tumor specimens and in urine sediments archived as cell blocks. In agreement with the few previous reports describing hTERT protein localization, we found hTERT to be localized to the nucleolus [13,30,31]. It is logical that hTERT is localized predominantly in the nucleolus, the site of nucleoprotein complex assembly, because hTERT is a subunit of the ribonucleoprotein telomerase enzyme [10]. In bladder tumor biopsy material we observed hTERT expression in cells adjacent to the tumor and expression was seen to diminish more distal to the primary lesion. This spatially associated hTERT positivity in apparently normal cells has been observed in other organs, including the colon [13], prostate [30] and melanocytic lesions [31]. The development of a reliable hTERT antibody has revealed this phenomenon and has the potential to better determine exactly where hTERT is expressed at the cellular level. The expression in normal cells could result from a number of possibilities, but the spatial relationship suggests that factors secreted from the tumor can induce hTERT transcription/translation in non-neoplastic cells.

Our goal was to evaluate the potential utility of hTERT immunodetection in urine sediments as an adjunct to cytology. Given the nature of the sample, which is often composed of scanty, dispersed cells, we did not attempt to estimate staining intensity or percentage of immunoreactive cells within the sample or block section. In this study, the presence (>3 cells observed) or absence of hTERT, specifically in transitional urothelial cells, was noted. The expression of hTERT was observed in 85% of the histologically confirmed bladder cancer cases, which represents a high rate of diagnostic sensitivity by examination of voided urine. Overall, the hTERT immunoassay demonstrated a positive predictive value of 77.8% and a negative predictive value of 75% in this study. The hTERT assay showed higher diagnostic sensitivity (84.8%) than published urine cytology data (~65%) for confirmed bladder carcinoma, however, the specificity of the hTERT immunoassay was 80%, lower than that obtained by cytologic evaluation (~90%). The specificity data do not support the use of hTERT immunodetection as a replacement for cytology, but the improved sensitivity of the assay suggests potential utility as an adjunct to cytology. It was notable that the majority of false-positive cases were found in the subset classified as cytologically atypical, but which have no evidence of disease to date. It will be interesting to see whether these patients will subsequently develop bladder malignancy in which case hTERT detection may have potential as an early detection or even predictive marker. However, specimens from patients with a history of bladder cancer are often deemed atypical cytologically, due to a 'field-effect', and in this study several cases were immunoreactive for hTERT. In agreement with telomerase activity assays in such patients [28], we did find hTERT-positive cells in patients who had no evidence of urothelial lesion at the time of sampling, but who did have a history of bladder cancer. Thus, it seems that hTERT detection may not be reliable in the monitoring of recurrent bladder cancer.

Our findings are in agreement with the previous telomerase-based studies in urological cancers, in that telomerase expression correlates with malignancy, but the use of immunohistochemistry adds specific advantages in the context of diagnosis. Although assays such as the TRAP assay or mRNA measurement can be designed to be semi- or fully quantitative, they do not provide information regarding the source of the protein. Immunohistochemical studies are beginning to reveal that normal cells can express hTERT under certain circumstances. In our study, normal urothelium adjacent to tumors expressed hTERT, as did apparently normal umbrella cells in some cases. Given the common presence of yeast in urine samples, it is easy to see how telomerase assays without morphological information could be misleading. Furthermore, the technical sensitivity of molecular assays may result in mislabeling samples which have only a few positive cells in a background of negative ones. With slide-based immunodetection, a single positive cell can be identified regardless of background content. These considerations are particularly relevant in samples where few cells may be present, such as urine sediments. When combined with cytology, the immunohistochemical detection of hTERT provides additional information upon which the observer can act. Bladder cancer detection based on the use of antibodies against tumor-associated antigens expressed by exfoliated urothelial cells reflects an attractive approach, because it is readily applicable using specimens obtained in a non-invasive manner and involves tissue processing and interpretation skills that are routinely available in a cytopathology laboratory.

## Conclusion

In conclusion, the detection of hTERT protein coupled with cytology has the potential to aid the diagnosis of benign and malignant bladder lesions in urine cytology specimens. Although the number of cytologic cases in this study was small, the results demonstrate the applicability of hTERT staining to fixed cytologic cell block samples. A larger study of the usefulness of hTERT applied to cytologic samples, including smear preparations, is in progress in our laboratory and will expand on these findings. Beyond that it may also be a useful marker to identify bladder carcinoma or other lesions that would be amenable to therapies that would involve interference of tumor proliferation through telomerase inhibition.

## Competing interests

The author(s) declare that they have no competing interests.

## Authors' contributions

WK performed cytology and pathology, co-designed the study and correlated clinical information with immunohistochemical data for all cases. SG conceived and designed the study, evaluated immunohistochemistry and drafted the manuscript.
